# Supplementary figures and images for: Genome-wide screen identifies new set of genes for improved heterologous laccase expression in Saccharomyces cerevisiae
Source: Microb Cell Fact. 2024 Jan 29;23:36. doi: 10.1186/s12934-024-02298-0 (PMC10823697; doi:10.1186/s12934-024-02298-0)

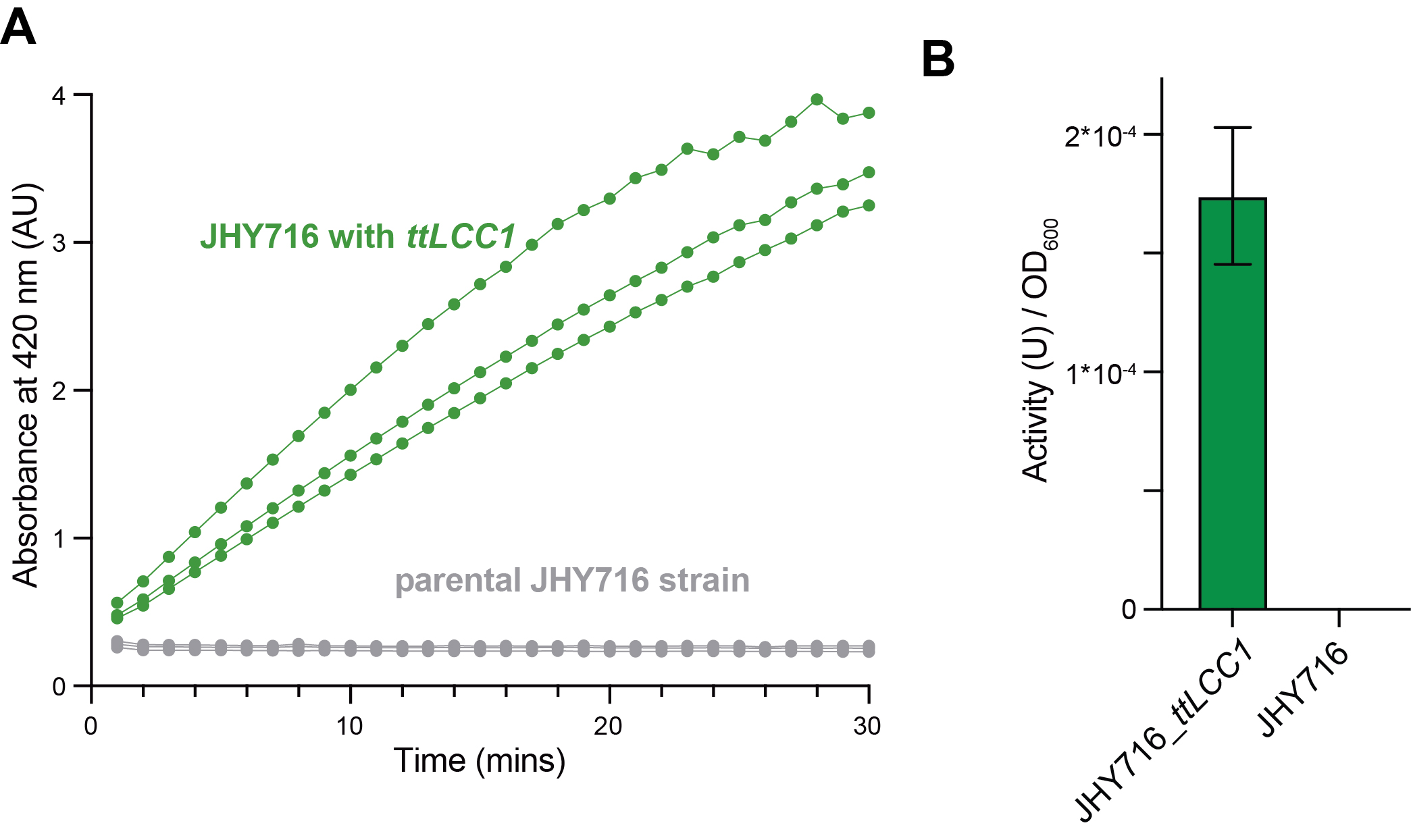

Supplement: Supplementary file 4 — Supplementary Material 4 [file 12934_2024_2298_MOESM4_ESM.jpg]

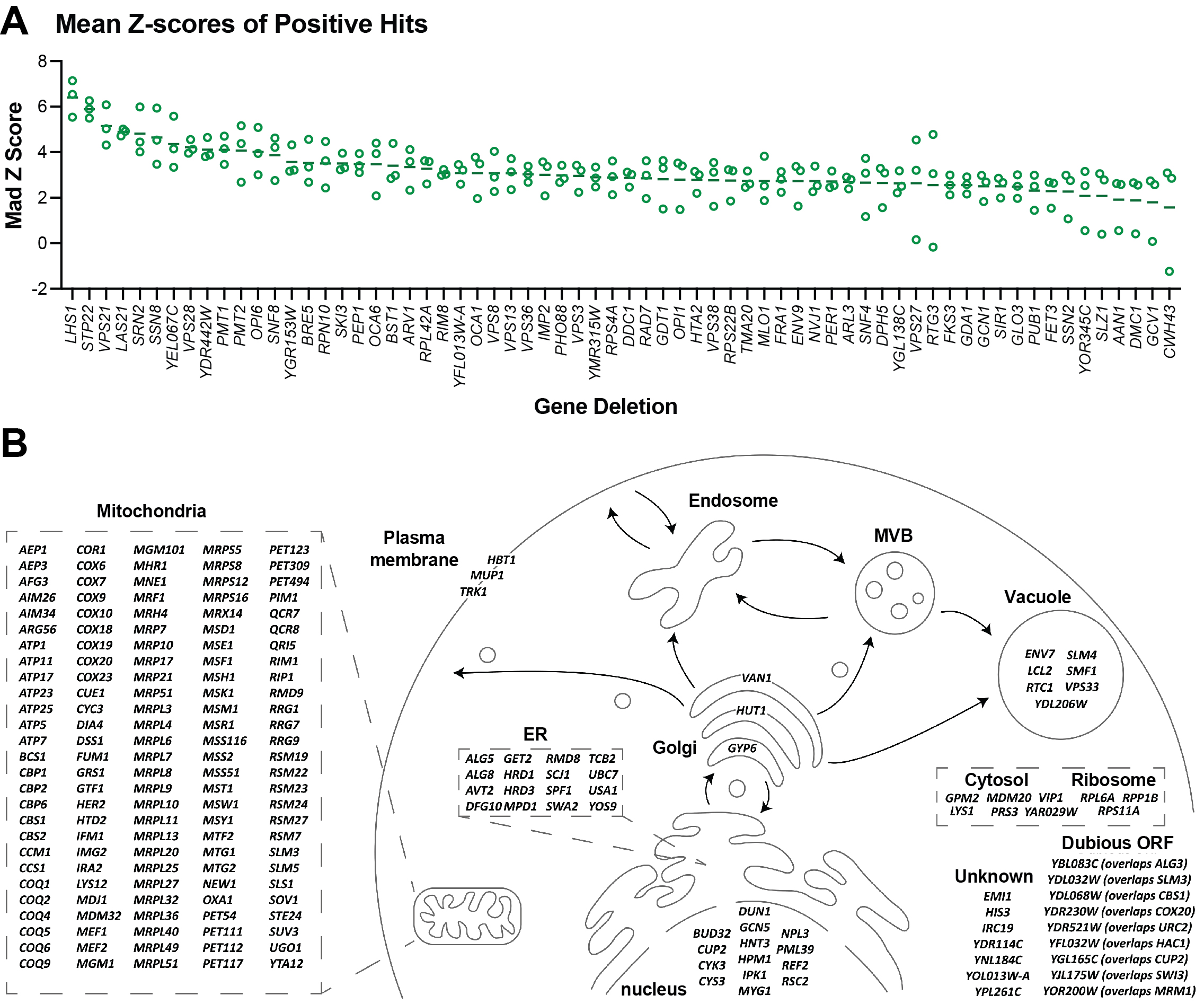

Supplement: Supplementary file 5 — Supplementary Material 5 [file 12934_2024_2298_MOESM5_ESM.jpg]

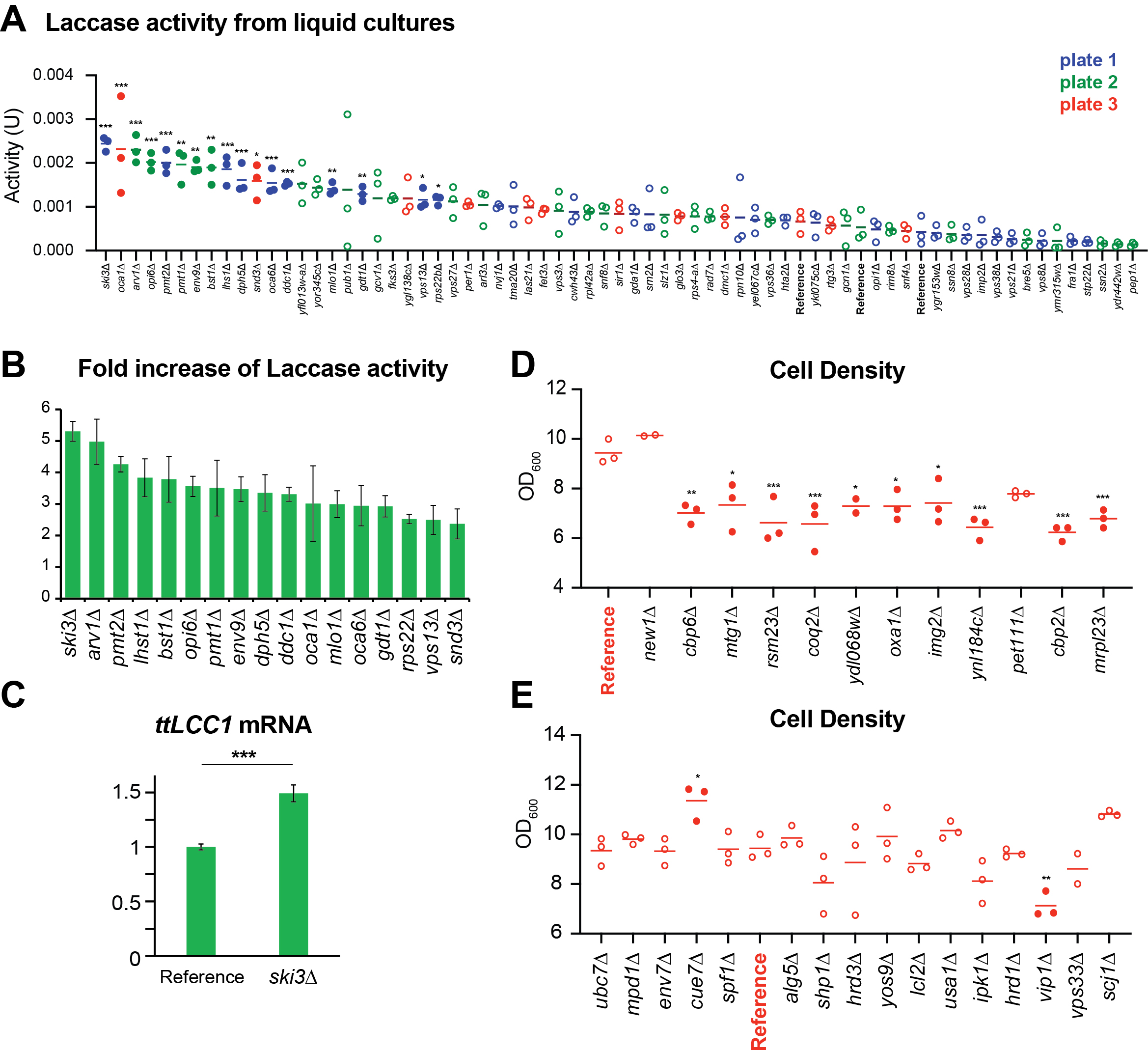

Supplement: Supplementary file 6 — Supplementary Material 6 [file 12934_2024_2298_MOESM6_ESM.jpg]

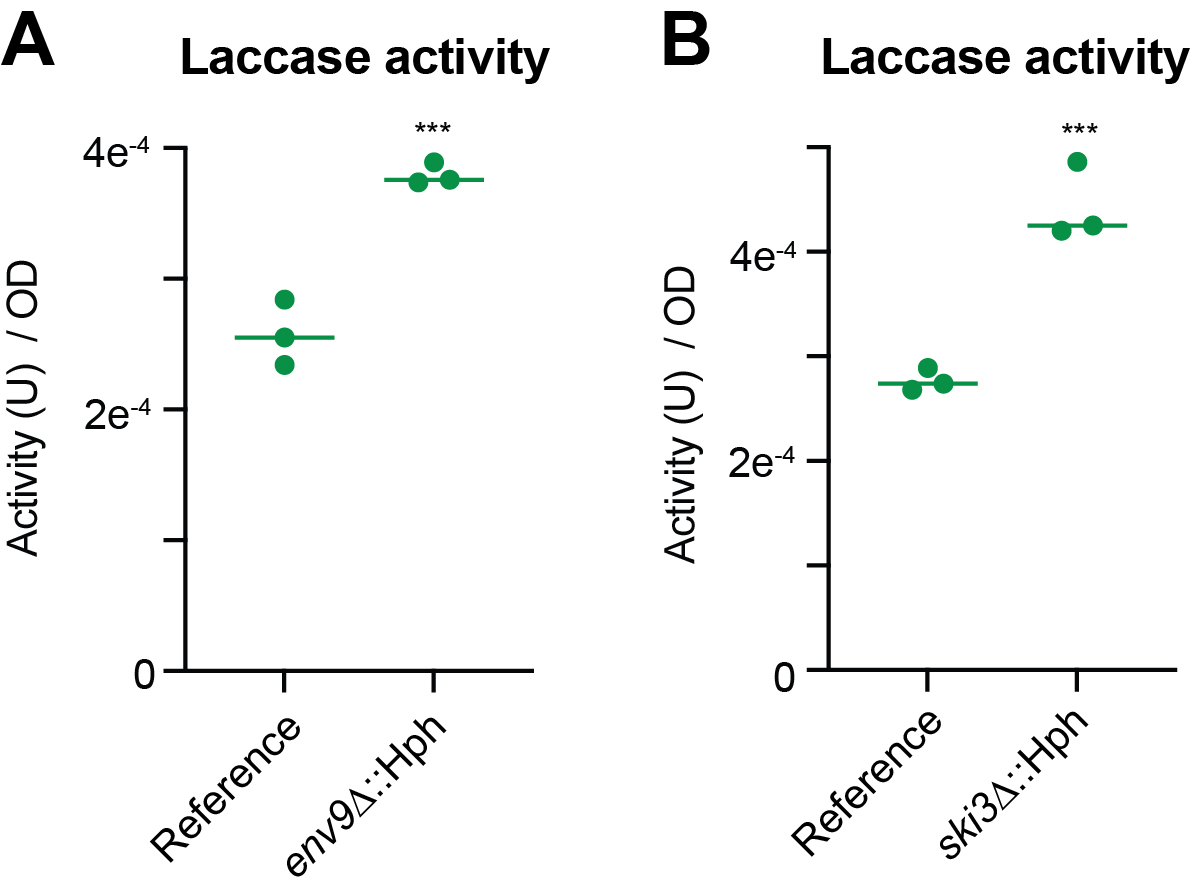

Supplement: Supplementary file 7 — Supplementary Material 7 [file 12934_2024_2298_MOESM7_ESM.jpg]
